# Mistletoe as an Anti-Hemorrhagic

**Published:** 1881-07

**Authors:** 


MISTLETOE AS AN ANTI-HEMORRHAGIE.—Our attention was called to the action of this old and familiar parasite, in an article by Dr. R. Lee Payne, Jr., in the *North Carolina Medical Journal*, as an oxytocic, and anti-hemorrhagie remedy of much value. A few days after perusing the article, an aggravated case of heæmoptysis came suddenly under our professional management and attention in the temporary absence of the attending physician. We were called at 12 o'clock at night, on the 31st of May, ultimo, to visit T. H. B., Esq., 35 years of age. We found him spitting and coughing up mouthfuls of florid arterial blood in rapid succession, and he stated he could feel the blood constantly welling up from the lower portion of the left lung. Having been apprised of his trouble before leaving home, and having received the day before a sample bottle of fluid extract of mistletoe (*Viscum Album*), it was taken along. We ascertained that the patient was taking fluid extract ergot, &c., in large doses, with little or no effect in lessening or restraining the hemorrhage. We gave him a half teaspoonful of the mistletoe, and in half an hour a teaspoonful more. In a few minutes after the second dose the color of the blood began to assume a darker hue, and soon after began coagulating and was expectorated in clots or lumps. The medicine was continued in half-drachm doses at intervals of two hours, until the following day, when it was so slight the patient was placed upon alternate doses of Squibb's fld. ext. ergot,and tinct. mur. ferri., 20 drops of each every three hours. The patient, though phthisical, has resumed his duties as a commission merchant—feeling better, he says, than he has done for years. And though one swallow may not make a summer, it is almost impossible to regard the prompt action of the anti-hæmorrhagic in Mr. Barnes' case as a mere coincidence. And it is therefore placed before our readers.

